# Study Protocol of Sleep Education Tool for Children: Serious Game “Perfect Bedroom: Learn to Sleep Well”

**DOI:** 10.3389/fpsyg.2018.01016

**Published:** 2018-06-26

**Authors:** Katie Moraes de Almondes, Maria E. M. Leonardo

**Affiliations:** ^1^Department of Psychology and Postgraduate Program, Postgraduate Program in Psychobiology, Federal University of Rio Grande do Norte, Natal, Brazil; ^2^Postgraduate Program in Psychology, Federal University of Rio Grande do Norte, Natal, Brazil

**Keywords:** children, sleep hygiene, sleep quality, serious game, sleep, health education, gamification, interactive learning

## Abstract

Promoting a healthy sleep is a big challenge and becomes a strategic priority in public health, due to the severe consequences on children’s development and risk to psychiatric diseases. Interventions that promote healthy sleep, such as those that focus on the dissemination of behavioral and environmental recommendations of sleep hygiene with children, are presented as an alternative. Serious game design offers wide-reaching domains in health applications and is increasing in popularity, particularly with children and teens because of it’s potential to engage and motivate players differently from other interventions. This study aims to evaluate effects of serious game on sleep hygiene recommendations “Perfect Bedroom: learn to sleep well,” on sleep habits and sleep parameters of healthy children. This is an experimental, prospective and quantitative study. We will randomize children in experimental (*n* = 88) and no intervention groups (*n* = 88). The experiment has four stages (pre-intervention, intervention, post-intervention, and follow-up), which will count with participation of children and their parents/guardians. In the evaluation stages, the guardians will answer questionnaires and scales to assess sociodemographic and health data, sleep habits and sleep pattern of their child. The children themselves will answer the following: a scale to assess sleepiness levels, a questionnaire to evaluate the serious game and the game itself, will characterize their bedroom and the activities they perform before sleep, with strategies developed by researches. Intervention with experimental group conducted with the serious game “Perfect Bedroom” will happen twice a week, for 3 weeks in a row, resulting in six sessions of 50 min each. Inferential analysis will be conducted for comparisons between groups and intragroups to measure effect of intervention in primary outcomes (sleep habits) and secondary outcomes (sleep parameters). We expect that the intervention with this game can provide valuable evidence to a new approach in promoting healthy sleep habits, with applications in clinical, educational, and familiar settings, which could diminish future health issues and risk at psychiatric diseases, decreasing the social burden of treatments for these conditions in children.

## Introduction

Sleep in children is a critical component for development and health, becoming an important call for action over the 21st century (Gruber et al., 2016). Sleep health is a “multidimensional pattern of sleep-wakefulness that promote physical and mental well-being,” and it includes seven potential domains that are considered in literature as predictors of health outcomes: Duration (total of sleep obtained in 24 h), Continuity (ability of falling asleep and returning to sleep when waking up without necessity), Timing (the time of sleep within the 24-h), Sleepiness/Alertness (ability to maintain attentive wakefulness); Quality (subjective evaluation of “good” or “poor” sleep); Regularity (consistency of sleep and wake times) and Rhythmicity (overall circadian rhythm pattern) (Buysse, 2014; Wallace et al., 2018).

This concept could be applied for children based on prior scientific evidences of negative consequences of children not getting enough sleep and/or poor sleep quality, such as irregularity in sleep schedules (Mindell et al., 2009; Matricciani et al., 2012; Kelly et al., 2013; Buysse, 2014; Paruthi et al., 2016). Literature data indicates association between deficits in these domains and negative outcomes, demonstrating critical role of sleep to physical, cognitive and psychological developmental process (Biggs et al., 2011; Boe et al., 2012; Simola et al., 2012; Turnbull et al., 2013; Brand et al., 2016; Gregory and Sadeh, 2016; Williams et al., 2016). A number of studies highlight evidences of consequences inappropriate sleep during development in presence of lower school performance, emotional and behavioral problems, decreased cognitive function (e.g., memory, attention, executive functions), health problems (e.g., increased risk of obesity, hypertension, diabetes), poor mental health and risk of psychiatric diseases in adolescence or adulthood (e.g., anxiety disorder, depression symptoms, oppositional defiant disorder, borderline personality disorder symptoms), lower quality of life and wellbeing (Kirov and Brand, 2011; Astill et al., 2012; Boe et al., 2012; Araújo and Almondes, 2013; Kelly et al., 2013; Shanahan et al., 2014; Csábi et al., 2015; Nelson et al., 2015; Chaput, 2016; Paruthi et al., 2016; Reidy et al., 2016; Lereya et al., 2017; Quach et al., 2018)

Additionally, secondary effects of such condition can be identified in the child’s family, as the following: negative impact on sleep quality, daytime functioning and subjective well-being; and the presence of depressive symptomatology, fatigue, stress, and conjugal conflicts (Meltzer and Mindell, 2007; Sadeh et al., 2010; Kelly and El-Sheikh, 2011). Similarly, children and teenagers’ sleep patterns is associated with parental sleep patterns and family functionality (Kalak et al., 2012; Bajoghli et al., 2013; Urfer-Maurer et al., 2017), especially on infants (Brand et al., 2009). This bi-directional association could be explained by: (1) influence of socioeconomic status (SES) in functionality and environment, (2) parenting style and psychological function of parents, and (3) correspondence of inadequate sleep habits between parents and their child (Bajoghli et al., 2013; Urfer-Maurer et al., 2017).

Insufficient and irregular sleep could be influenced in part by poor sleep hygiene (Mindell et al., 2015; Brockmann et al., 2016; Brambilla et al., 2017). Study with caregivers reports indicate that sleep hygiene is associated with how well children ages 0–10 years sleep (Mindell et al., 2009). Sleep hygiene practices includes a set of behavioral and environmental recommendations on sleep environment, sleep routine and daytime activities with the aim of promoting healthy sleep (Irish et al., 2015).

Common sleep hygiene practices in the sleep environment include providing a comfortable bedroom, without excessive noise or distracting stimuli, and with adequate temperature and luminosity. Promotion of a sleep routine is significantly important for children, as it helps to maintain consistent sleep schedules on school days and weekends, and to avoid sleep deprivation. Additionally, a bedtime routine could be composed by activities that help prepare the child to fall asleep, such as: reading, talking about the day, bath time and story-telling (Irish et al., 2015; Allen et al., 2016).

The National Sleep Foundation’s recommendations on sleep duration indicate that school-age children, focus of this study, should sleep in between 9 and 11 h. Still, in this age it is observable widespread use of technology, with increased use of mobile devices (Hirshkowitz et al., 2015; Brockmann et al., 2016). There is as well an increasing demand of their time on school, extracurricular and social activities (Kelly and El-Sheikh, 2011).

Several studies discuss that exciting activities and use of video devices near bedtime (e.g., television, tablet, video game, smartphones), an uncomfortable sleeping environment, and/or irregularity of bedtimes and waketimes are related to sleep onset-delay, shorter sleep duration, later bedtime and increased daytime sleepiness (Mindell et al., 2009; Cain and Gradisar, 2010; Hale and Guan, 2015; Allen et al., 2016; Brambilla et al., 2017).

While literature reports these negative effects of poor sleep hygiene, there is still difficulty to translate research in pediatric sleep into effective actions and policies that may change this condition. Recent manuscripts identify just 15 studies aiming to improve sleep of children and teenagers (Gruber et al., 2016; Blunden, 2017). Sleep interventions have been applied in family, school and clinical contexts, involving parents, teachers, and children, resulting in improvements in knowledge about sleep and promoting few changes in sleep behaviors (Wilson et al., 2014; Blunden and Rigney, 2015; Ashton, 2017; Blunden, 2017).

The intervention “Sweet Dreamzzz Early Childhood Sleep Education Program^TM^” was conducted with teachers, parents, and pre-school children. During eight school days children received lessons about the following topics: bedtime routine and importance of sleep before 08:00 pm. The researchers used recreational resources, such as: one bear named Teddy, stories about sleep and one BINGO. This intervention improved the parent’s knowledge about sleep, attitudes and self-efficacy immediately post-intervention, without lasting effects. As secondary outcomes, visible improvement was only in sleep duration, with a 30 min increase; however, said increase was evaluated through subjective measures in sleep diaries answered by parents, and the children’s knowledge has not been assessed (Wilson et al., 2014).

Other study, also involving teachers, parents and children improved in 10 min sleep duration of children with ages between 11 and 13 years but did not identify changes in sleep knowledge and sleep habits. In this study, lessons were delivered with Microsoft Power Point Presentations about sleep physiology and hygiene. Similar to the study of Wilson et al. (2014) the assessment of sleep behavior was not objectively measured and the classroom attendance was not evaluated, which could indicate different levels of exposition of sleep education program (Rigney et al., 2015). Along with intervention on children’s health, there are sleep training interventions in clinical contexts that aim to improve emotional, behavioral, and/or social performance through healthy sleep. Keshavarzi et al. (2014), developed a 12-week sleep training intervention for children with attention deficit/hyperactivity disorder (ADHD), which included recommendations on sleep hygiene and orientations to define both sleep and wake-up times, to gradually improve sleep duration. Results from this research showed improvements on sleep, mood, and relationships with familiar and friends.

There are also interventions that include just parents. Mindell et al. (2016) examined efficacy of a sleep hygiene educational campaign “Sleep Well” directed to parents and developed with families of low SES assisted by One House at a Time’s Beds for Kids, which provides beds for children of these families. Thus, the study had two groups: the sleep education group received the following three sleep messages in three objects (sheet, on a bookmark, and on a refrigerator magnet): (1) have a bedtime before 09:00 pm, (2) avoid caffeine, and (3) keep electronics out of the bedroom. The control condition group received a packet with information about dental education. Results from this research indicate that alterations in sleep environment were associated with the increase in sleep duration and reduction in electronics devices present in the bedroom for all children, but the intervention group showed more benefits in the decrease of electronics devices in bedroom and extension of nocturnal sleep (Mindell et al., 2016). The relationship between sleep and lower SES has been explored, with children from lower income and/or maternal education families experiencing sleep problems, such as difficulties in initiating and/or maintaining sleep; and a shorter time in bed (Boe et al., 2012).

Another intervention with parents in a pediatric primary care clinic verified that a simple, punctual and inexpensive educational tool, such as one brochure, may improve parent’s knowledge about healthy sleep in childhood, as well as increase the number of caregivers that planned to change inadequate sleep habits for children (Jones et al., 2013). However, this research also did not provide an objective measure of children’s sleep, since self-report intentions to improve sleep could be based on social desirability by the parents.

These studies present intervention proposals with limitations. First, sleep patterns assessment in most cases is by subjective measures and answered by parents. Recent reviews indicated that the caregiver’s knowledge about sleep needs and routine is poor (McDowall et al., 2017); the parental perception of sleep amounts is complex and limited to parental perception of children’s sleep behaviors, which could influence in sleep diaries accuracy (Jones et al., 2013; Sadeh, 2015).

Ecological assessment of the children’s knowledge about interventions themes in baseline and post-intervention was not included in any of the researches, but one study used a Sleep Knowledge Questionnaire to assess this parameter (Rigney et al., 2015). Furthermore, just one study used non-randomized trials to allocate school and children in intervention or control conditions.

Therefore, there is a big gap in the interventions described, which is the inclusion of a motivational approach to increase interest and involvement of participants, especially the children themselves, as several interventions only receive information from parents. In view of this gap and the increase in use of serious games on: (1) health, with the purpose of promoting quality of life and healthy habits; (2) cultural training; (3) professional learning and training; (4) social skill training; (5) to support and help decision making and (6) to teach, motivate and help in formal education (Blumberg and Burke, 2014; Calderón and Ruiz, 2015; Boyle et al., 2016; Mahlmann et al., 2017), our research team proposes that it may be used as an integrable tool to Sleep Hygiene, in order to include the fantasy and playful components in this type of intervention for children.

The main objective of this type of game is to promote formal and informal health education, in addition to behavioral modification (Blumberg and Burke, 2014). It can be classified according to the application platform, as digital and non-digital. The first category involves human interaction with user interfaces from any electronic devices (cell phone, tablet, computer), which promote user feedback, while non-digital users interacts with other resources, such as boards, cards, papers, and pencils (Mitamura et al., 2012; Petri and von Wangenheim, 2016).

Previous research indicated the potential of serious games for the promotion of a healthy lifestyle, suggesting that serious gaming interventions alone or with an educational module have a positive effect in these parameters (DeSmet et al., 2014; Lau et al., 2017). There are games that aim to promote healthy eating, regular physical activity, safe sexual behavior and the management of chronic illnesses (Scarle et al., 2011; DeSmet et al., 2015; Viggiano et al., 2015). On the same vein, interventions with exergames (active videogames) have been used also to improve behavior, physical skills and in rehabilitation (Navarro et al., 2017). This could promote benefits in others parameters, such as sleep (Mahlmann et al., 2017). However, even with the wide dispersion of intervention using games, there are still few studies that validate the effectiveness of this type of intervention (Moreno-Ger et al., 2014).

For children, serious games are an ideal approach and can become a promising intervention modality, since playing games is a popular leisure for the new generations. The idea is that a serious game could enhance engagement by features of game design, types of interaction and user’s experience (entertainment), which provide positive experiences that maintain the player’s involvement and increase exposure of health intervention (Baranowski et al., 2016).

In this way, our research team developed a serious game based on recommendations of Sleep Hygiene, named “Perfect Bedroom: learn to sleep well.” Considering the potential benefits of these approaches taken together, we hypothesized that an intervention with the serious game “Perfect Bedroom: learn to sleep well” may be helpful to modify: (a) inappropriate sleep habits related to irregular sleep and wake times, (b) inadequate sleep environment and pre-sleep activities, and consequently (c) improve sleep parameters, such as sleep duration and sleep efficiency, and decreasing sleep latency somnolence levels.

Therefore, an experimental, prospective and quantitative study will be used to compare effects of game application by in change of habits and parameters of sleep, measured by objective and subjective tools, as well as assessing the player’s decisions as a result of playing this serious game, with the inclusion of an ecological assessment about sleep routine and bedroom organization by children. As far as our knowledge goes this is the first intervention that included a serious game to promote healthy sleep habits to children.

## Materials and Equipment

Exclusion criteria for participants will be as follows: (1) less than 7 years and more than 9 years old; (2) use of psychoactive drugs; (3) absent from interventions (in which cases data from this participant will be excluded from analysis, but they will continue to participate in the intervention) and (4) diagnose of sleep disturbance or any health problems, such as neurological disorders, and psychiatric, cardiovascular or genetic diseases. Thus, to characterize the sample and to verify inclusion criteria questionnaires and scales will be applied, and strategies developed by researchers with parents/guardians and their children. These measures and the serious game itself are described below.

### Measures

#### Sociodemographic and Health Questionnaire

This questionnaire was designed to collect information about health, daily activities and demographic characteristics from parents and their children. It will be responded by parents and contains questions on:

(1) Child data: birth date; age; gender; grade level; potential sleep disorders, present or past; child’s medical and psychological condition; use of any medication or treatments; and additional activities of children.
(2) Family data: job and education level of both parents, marital status, number of family members living with child and number of brothers/sisters.

#### Socioeconomic Status Questionnaire

The questionnaire allows inference of the family income and stratifies the population into six economic classes (A, B1, B2, C1, C2, D-E). This criteria was established by the Brazilian Association of Research Companies (Associação Brasileira de Empresas de Pesquisa – ABEP) and considers: possession of comfort/high value objects (washing machine, car, freezer, etc.), access to public services (e.g., potable water) and the level of education of the family member who contributes most to family income (Associação Brasileira de Empresas de Pesquisa [ABEP], 2014).

#### UNESP Sleep Habits and Hygiene Scale (Child Version)

This scale contain 16 questions and will be used to evaluate characteristics of children’s sleep, according to four indicators that are considered causal to sleep hygiene: sleep routine (activities realized before bedtime); physiological alert (diurnal habits that would promote excitement or physical discomfort near to bedtime); cognitive/emotional aspects (indicators of emotional regulation and wellbeing before bedtime) and sleep environment (adequate sleeping environment in terms of temperature, comfort, and organization) (Pires et al., 2012). Each item is assessed on a five point of frequency scale from “never – no day” to “always – every day.” Furthermore, the scale contains five questions to evaluate bedtime resistance and daytime somnolence, assessed also on a five-point scale of frequency. Higher scores indicate worst habits of sleep hygiene. This has been empirically validated for use in Brazil.

#### Sleep Disturbance Scale for Children

This scale was elaborated by Bruni et al. (1996) and validated to use in Brazil. It is composed by 26 items, with a self-report rating that assesses sleep disorders in children, answered by parents. The scale has six factors: (1) disorders of initiating and maintaining sleep; (2) sleep breathing disorders; (3) disorders of disturbance and nightmares; (4) sleep wake transition disorders; (5) disorders of excessive somnolence; and (6) sleep hyperhidrosis. Each item is assessed on a five-point scale from “never” to “always” and higher scores indicate more sleep problems (Bruni et al., 1996; Nascimento-Ferreira et al., 2016).

#### Sleep Diaries

Sleep diaries are an extremely helpful tool to assess sleep patterns (Carney et al., 2012). We adapted a model of a diary and each page contains questions about children’s sleep for each day, forming a booklet that will be answered by the parents over 10 days. It will provide information about: (1) sleep and wake-up times, (2) sleep latency (amount of time taken from bedtime until the child falls asleep), (3) number of awakenings during the night, (4) total sleep time, and (5) naps timing and duration. Additionally, it was included questions on: activities before bedtime, how the child woke up and why; and parent’s perception of wellbeing of their child after each night of sleep. These measures will be registered over the 10 nights, allowing the researchers to examine the children’s habitual sleep patterns in weekdays and weekend.

#### Actigraphy

Actigraphy will be used to objectively measure sleep/wake behavior, using the Actiwatch model AT5030 (Condor Instrument, São Paulo, Brazil). This device measures activity, temperature and exposure to light. Taken together, these measures and the information from sleep diaries can be used to properly assess sleep and wake patterns of children. Actiwatches will be used by children on their non-dominant wrist over 10 days and while parents will be responding sleep diaries. Child and parents will be informed about the guidelines for actiwatch use, including always using the actiwatch, except when engaging in contact sport, bathing, or swimming, and to not apply perfume or moisturizer near the device. Further, the use of the event marker will be encouraged to indicate bedtime and wake time. Additional information was included in the sleep diaries to reinforce how to use the device. The following variables will be derived from the actigraphy records: (a) total sleep time (the total of sleep obtained during a sleep period); (b) sleep latency (amount of time taken from bedtime until the child falls asleep), and (c) sleep efficiency (i.e., number of hours slept/number of hours spent in bed × 100) (Markovich et al., 2015).

#### Strategy “Set Up Your Bedroom”

**FIGURE 1:**
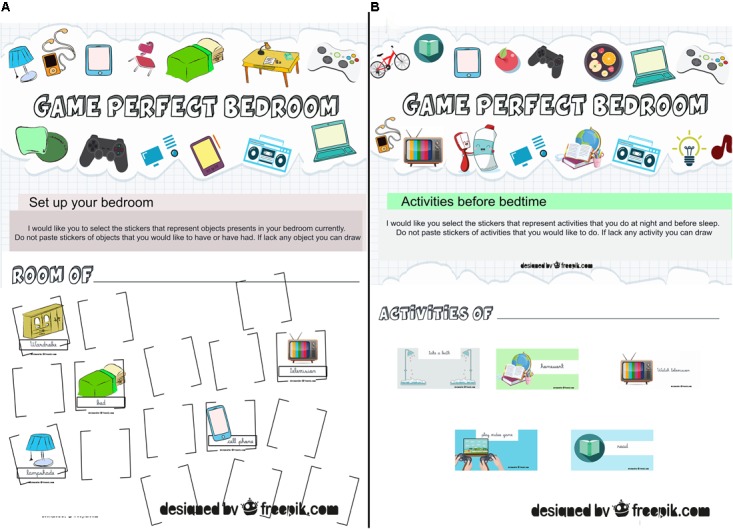
Strategies developed by researchers by a database of free images (freepik.com) to evaluate what the users will be able to do as a result of playing serious game. **(A)** Strategy “Set up your bedroom.” **(B)** Strategy for mapping activities before bedtime. Figures adapted from freepik.com by researchers and respecting terms and conditions to use.

This strategy was created by the researchers to characterize a child’s bedroom according to their perception. Each child will receive one kit with one sheet of paper A4 to set up your bedroom and 17 stickers that represent common objects that could be in bedrooms, such as: bed, wardrobe, pillows, lamps, video game, cell phone, television, etc. (**Figure 1A**). After that, they will read and apply the following instruction: “I would like you to select the stickers that represent objects presents in your bedroom currently. Do not paste stickers of objects that you would like to have or have had. If the stickers lack any object, you can draw it.” In addition, this strategy will be used to measure what the child knows or is able to do as a result of playing the serious game “Perfect Bedroom: learn to sleep well” (Calderón and Ruiz, 2015).

#### Strategy for Mapping Activities Before Bedtime

This strategy was also developed by researchers and assesses the activities that the child usually does at night and before sleep. Each child will receive one kit with one sheet of paper A3 and fifteen stickers that represent common activities that they could do before sleep at night, such as: take a bath, play video games, brush their teeth, watch TV, read, listen to music, do homework, turn off the lights (**Figure 1B**). After that, they will read and apply the following instruction: “I would like you to select the stickers that represent activities that you do at night and before sleep. Do not paste stickers of activities that you would like to do. If the stickers lack any activity you can draw it.” Similarly to the strategy described above, this one will be used to measure the knowledge the children have and what they are able to do as consequence of playing serious game (Calderón and Ruiz, 2015).

#### Pictorial Sleepiness Scale of Maldonado

Sleepiness levels will be assessed by an adapted version (Belisio, 2014) of the Pictorial Sleepiness Scale of Maldonado (Maldonado et al., 2004). It will be applied during six consecutive class days between 02:00 pm and 03:00 pm, following class schedule. The data collected in the first day will be excluded from the analysis to decrease effects of a new person in the classroom. In the application the children will be requested to indicate the face on the scale that best represented how they were feel at the time, that is, if they felt sleepy or not. Higher scores indicate elevated levels of somnolence.

#### Evaluation of Serious Game “Perfect Bedroom: Learn to Sleep Well” by Children

For the purpose of allowing the children to evaluate the serious game, we developed a questionnaire with textual and graphics elements. It includes quality characteristics that current literature indicate to be relevant to evaluate serious games (Calderón and Ruiz, 2015) and contains questions on:

(1) User’s satisfaction and user’s experience, to evaluate their attitude and behavior related to the game.
(2) Understandability, to assess the ability of the serious game of being understood.
(3) Enjoyment, to assess if the game provides a fun experience to the users.
(4) Learning outcomes, to identify what the users will be able to do as a result of playing this serious game. These measures will be provided from comparisons between the results from pre and post-intervention of the strategies “Set up your bedroom” and “Mapping activities before bedtime.”

## Stepwise Procedures

### Participants

This study aims to recruit 176 healthy children enrolled in two private schools from Natal (Rio Grande do Norte – Brazil), as the number of children per class in these educational institutions ranges from 23 to 28. Schools will be selected by convenience, as there are difficulties to promote parental and school involvement (Jones et al., 2013; Wilson et al., 2014; Mindell et al., 2016), due to the concern of staff and parents about the activities developed in school and the necessity to adjust the research schedule to that of school activities, such as evaluation periods, vacation, science fair or school games. Furthermore, sample size was carried out using G^∗^Power 3.1.9, with alpha level of 5%, effect size of 0.5, and power of 95% for the two groups (Faul et al., 2007; Aberson, 2015). Results from this analysis indicated 88 participants to each group.

### Design and Procedures

It will be used an experimental, prospective, quantitative and randomized controlled trial to compare effects of game application on change of habits and parameters of sleep, besides what the users will be able to do by playing this serious game. In order to achieve that, the following steps will be conducted: (1) randomization of participants to allocate each one in experimental or control group; (2) use of a control group that should not be submitted to intervention, to infer highest internal validity; (3) intervention with experimental group, which will allow us to evaluate if there was any modification in the parameters evaluated, determining the validity of the intervention and (4) evaluation of participants at three distinct moments throughout the study, namely: pre-intervention, immediate post-intervention and follow-up assessment, after 4 weeks (Suresh, 2011).

After selecting the schools by convenience, experimental and control groups are selected by two processes of randomization. The first will select which classes will compose the experimental and control groups in each school, throughout a basic method of randomization that is flipping a coin; in other words, one situation will be associated with each side of coin (e.g., heads – control and tails – experimental) to determinate the assignment of each class. Second, the function “Randomly assign subjects to groups” in online software GraphPad^1^ will be used to allocate participants of each class with valid data for analysis to each group.

Upon enrolment, the four stages of research will be followed, counting on the participation of children and their parents/guardians. All encounters with parents/guardians will happen at 6:40 pm, after the children’s classes, due to the parent’s convenience. First, during the stage of pre-intervention, questionnaires and scales will be applied to assess sociodemographic data, and characterize sleep habits and sleep pattern, by filling out the UNESP sleep habits and hygiene scale (child version) and sleep diary, besides giving the actiwatch to be wore by the children. Parents will also answer the Sleep Disturbance Scale for Children to identify the presence of sleep disorders. After this, parents from experimental group will participate in a workshop conducted by the research team about sleep habits and the importance of healthy sleep in childhood. The team will be available for an additional encounter 1 week after evaluation to clarify doubts about filling in the sleep diary and/or use of actiwatch.

The children themselves will be active in the evaluation steps. All evaluations with them will occur on the first Monday after each meeting with parents, once research begins with the parent’s authorization. In this stage, the children will answer the Pictorial Sleepiness Scale of Maldonado to evaluate sleepiness levels and will characterize their bedroom and the activities that they do before sleep at night, using, respectively, the strategies “Set up your bedroom” and “Mapping activities before bedtime.”

After pre-intervention evaluation, the experimental group will be submitted to the intervention with our serious game “Perfect Bedroom: learn to sleep well,” which will be conducted by three researchers during the children’s classes, at 02:00 pm on Tuesdays and Thursdays (see section “Intervention”).

The control group will follow the institution’s school activities defined on the school schedule. The only moments of contact with study staff will be during evaluation, pre-, post and following intervention, to assure a proper control condition, in accordance to our research question about intervention works (Holmes et al., 2018). Considering this, further control will be needed for the following factors: contact with research team limited only to evaluation periods to avoid confusing factors such as attention, warmth, or human relationships. In the evaluation stages, the guardians will answer questionnaires and scales to assess sociodemographic and health data, sleep habits and sleep pattern of their child. The children themselves will answer: a scale to assess sleepiness levels, a questionnaire to evaluate the serious game and the game itself, will characterize their bedroom and the activities they perform before sleep. Also, teachers will be informed only essential information about the study and will be instructed to not use the knowledge acquired about sleep in class during the period of the research.

**FIGURE 2:**
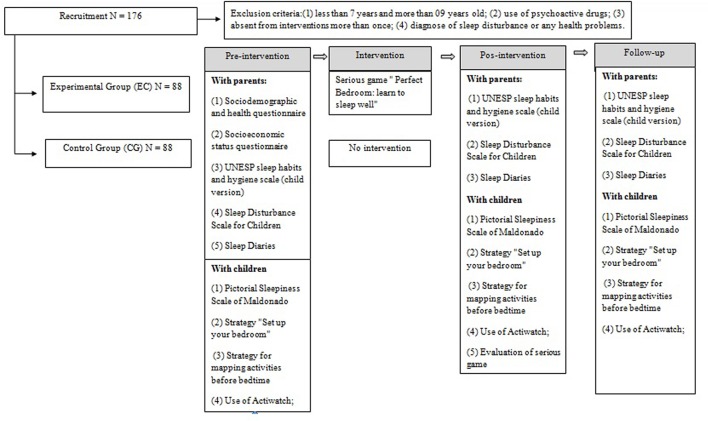
Flow chart of design and procedures.

After completion of the intervention, the last two steps, post intervention evaluation and follow-up evaluation, will be conducted after 4 weeks. In these stages sleep habits and sleep patterns will be re-evaluated by the parents. Additional encounters 1 week after each evaluation will be provided to clarify any doubts about filling in the sleep diary and/or use of actiwatch. During these steps the children will be required to answer the Pictorial Sleepiness Scale of Maldonado and to characterize their bedroom and any activities that they perform before sleep at night (**Figure 2**). Thus, there will be three meetings with parents/guardians during evaluation steps, with three more other encounters to elucidate any doubt about assessments. Missing data will result in individuals being excluded without outcome.

After completion of all evaluation stages, workshops about sleep habits and the importance of sleep in childhood will be conducted by research team with teachers of the schools that participated in the study.

### Intervention With Serious Game “Perfect Bedroom: Learn to Sleep Well”

The serious game “Perfect Bedroom: learn to sleep well” will be the main resource used in this intervention, and was developed by Katie Moraes de Almondes (Associate Professor at the Department of Psychology and on the Postgraduate Program in Psychobiology), Maria Emanuela Matos Leonardo (Master’s Student on the Postgraduate Program in Psychology), and Fransueldo Florêncio Ribeiro do Ó (Student on Science and Technology). It is currently in process of patenting, registration number BR 10 2015 032215 1, and copyright has been registered in Brazil National Library Foundation, registration number 681. 074.

The game is based on sleep hygiene and includes a series of behavioral and environmental recommendations to promote healthy sleep, with the aim to teach and promote good sleep habits for children. In this regard, our serious game provides explanations about sleep hygiene habits, presenting them in a playful way, to make it more interesting for children. It includes the challenge of mounting a “perfect bedroom” based on the application of knowledge of healthy sleep habits.

**FIGURE 3:**
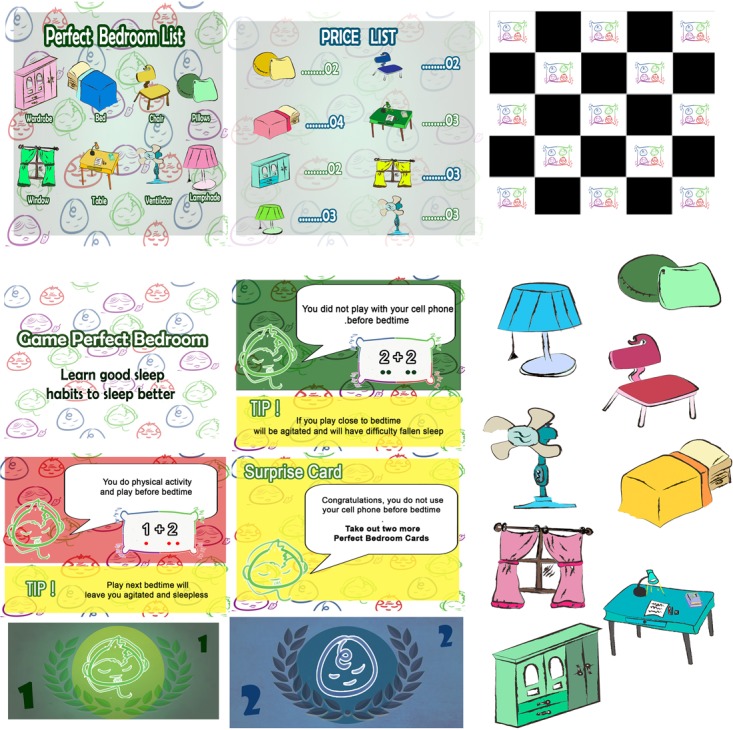
Components of serious game “Perfect Bedroom: learn to sleep well.” Copyright registered in Brazil National Library Foundation, registration number 681. 074.

Serious games such as this are composed by four components: boards, cards, bank notes and objects (**Figure 3**). First, to indicate adequate and inadequate sleep habits, cards with recommendations of sleep hygiene and additional explication about importance or consequences of do or not do this action (e.g., You do physical activity and play before bedtime. Playing next to bedtime will leave you agitated and sleepless) will be used. These cards also have indications of rewards for good sleep habits, that will be paid in Sleep Perfect’s bank notes. On the other hand, when cards have inadequate sleep habits no rewards will be given.

Indicative phrases of adequate and inadequate sleep habits were elaborated according to findings in Allen et al. (2016) and Mindell et al. (2009). In this first research, evidences of common sleep recommendations were investigated to promote healthy sleep in children, identifying strong empirical evidences to support schedules and bedtime routines; limited access to electronics before bedtime and in bedroom; and independence to fall asleep at bedtime and when waking up at night. Moderated or limited evidences were found about: consistency in daytime routine; environment and conditions of bedroom (e.g., quiet, dark and with comfortable bed); or eating habits (e.g., to limit caffeine consumption; not to go sleep hungry; eat a healthy balanced diet) (Allen et al., 2016).

Another study that we used examined association between sleep hygiene and how well children sleep (Mindell et al., 2009). This choice was based on the key issue that it is essential to understand the relationship between adequate sleep habits and sleep patterns to propose interventions in habits that influence sleep. Thus, in school-aged children, target population of this protocol, significant associations were verified between presence of parents in bedroom, late bedtime, consumption of caffeine, television in bedroom and reduced total sleep times and more night waking. Thus, the research team selected recommendations according to empirical evidence of sleep hygiene practices that could promote healthy sleep.

The second component of the serious game are four boards representing the floor of the bedroom that the children will assemble. In addition, to be more interesting and dynamic we included bonus cards (e.g., Congratulations, you do not use your cell phone before bedtime. Take out two more Perfect Bedroom Cards!) and surprise cards (e.g., You have television and computer in your room. These objects hinder the beginning of sleep! Pass the turn!). To allow the players to buy the items that will compose the bedroom bank notes with values of 1 and 2 were created. Available for the children to buy with the notes are 32 objects, divided in eight types (lamp, pillow, bed, chair, window, table, wardrobe, and fan), with four different colors (pink, yellow, green, and blue), to assemble the ‘perfect bedroom’ without electronics devices. The last pieces of the game are cards that list the objects that can be purchased and their values.

To play, all participants will receive the necessary materials described, with the starting player randomly chosen. The first participant will draw a card from the perfect room cards deck, which contains adequate (positive cards) and inadequate (negative cards) sleep habits, bonus or surprise cards. After drawing one card the player must do the card indicates. The player who wins is the one who assembles the perfect room first, with all the items on the list, or the one with the most objects in their board.

The intervention will be conducted by three previously trained researchers. It will occur at the children’s school, twice a week (on Tuesdays and Thursdays), starting at 02:00 pm, for 3 weeks in a row, resulting in six sessions of 50 min each. In each session players will be challenged to set up the “perfect bedroom.” They will apply the behavioral and environmental changes of sleep hygiene to promote healthy sleep.

### Ethics Statement

This study was approved by the Research Ethics Committee of the Federal University of Rio Grande do Norte, Brazil (registration number 62016916.2.0000.5537). All participants (children and parents/guardians) will be volunteers and will provide written informed consent to their participation in the study. The study was conducted in conformity with the ethical standards proposed in the World Medical Association Declaration of Helsinki. In addition, the control group will receive the intervention after all evaluation stages.

### Proposed Analysis

The software IBM (Statistical Package for the Social Sciences [SPSS], 1968), version 21.0 will be employed for data analysis, assigning the 5% significance level for all statistical tests. The Kolmogorov–Smirnov (KS) Test will be used to verify normality. Inferential and descriptive statistics will be used to test hypothesis.

### Controlling for Covariates

To identify differences between experimental and control groups, which could invalidate the comparison procedure, inferential analyses in demographic and sleep variables will be conducted to test homogeneity. Adequate tests will be selected according to variable’s type and assumption or not of normal distribution of data in SW, as following:

(1) A Chi-square test (χ^2^) and generalized Fisher test will be used for categorical variables;
(2) An independent student’s *t*-test (*t*) will be used for continuous variables.

### Intergroup and Intragroups Analysis

Once confirmed that the groups did not present significative differences, the comparison procedure will be conducted. Variables included in sleep patterns (sleep latency, sleep efficiency, sleep and waking up times, total sleep time), sleep hygiene and sleep problems will be considered dependent variables and will be compared between experimental and control groups, during the three points in time where they will be evaluated, using independent student’s *t*-test and chi-square (χ^2^) with odds ratio and dimension of association (Phi and Cramer’s V), depending on the nature of the data. It will allow us to identify if the intervention with the serious game yielded improvements in objective sleep measures and sleep hygiene.

To evaluate possible improvements intragroup, comparisons will be conducted using: ANOVA (F) for repeated measures with Bonferroni’s post test and Q Chochran test. For all numeric variables percentage will be calculated with delta differences (post-test score minus the pre-test score) and percentage’s change [(post-test score) minus (pre-test score) divided by (pre-test score) multiplied by 100]. These measures will allow us to evaluate the intervention’s immediate effects and the maintenance of this effect after 4 weeks.

### Evaluation of Serious Game “Perfect Bedroom: Learn to Sleep Well”

The statistical analysis will involve analyzing the responses from the evaluation questionnaire and strategies developed by the researchers (“Set up your bedroom” and “Mapping activities before bedtime”). For the questionnaire descriptive analyze and exploration of frequency will be conducted with crosstabs command. It will allow us to characterize the children’s perception of the game in the following aspects: (a) user’s satisfaction and user’s experience; (b) understandability; and (c) enjoyment. These indicators will be collected in a typical Likert scale with a five-point ordinal scale that represents five conditions in binary data (I did not like play = 1; I did like play = 2). In addition, the software package Iramuteq will be used for the qualitative analyze of the open question “How it was to play the game Perfect Bedroom: learn to sleep well?”).

The final point of the evaluation, learning outcomes, will be evaluated with inter and intragroup analysis of the strategies cited below. The activity of mapping bedroom responses will be categorized in binary data (object absent in bedroom = 1; object present in bedroom = 2) and the total of inappropriate and suitable objects present in the bedroom will be counted. Attribution in each group will occur according to previous studies indicating the negative effect of electronic devices in children’s room (Cain and Gradisar, 2010; Cespedes et al., 2014). The same will be for the analysis of the strategy for “mapping activities before bedtime.”

Inferential tests to intragroup and intergroup will be carried out in binary and numeric data, respectively, ANOVA (F) for repeated measures with Bonferroni’s post test and Q Chochran test to intragroup; and independent student’s *t*-test (*t*) or Chi-square test to intergroup analysis.

### Additional Exploratory Analysis

The relationship between sleep measures, sleep habits and demographic data will be evaluated with Pearson (r) correlations. Those will provide information to prove the hypothesis that acknowledgment and application of adequate sleep habits may improve sleep on children. Furthermore, it will allow us to identify if any demographic data (e.g., family incomes, education levels) is related to sleep parameters and sleep habits.

## Anticipated Results

Considering the constellation of significant and negative consequences of sleep alterations for children’s overall quality of life, the objective of this protocol of study is to verify potential effects of the serious game “Perfect Bedroom: learn to sleep well” in improving sleep hygiene habits, which includes recommendations in sleep environment, sleep routine, and daytime activities, and, consequently, the promotion of better sleep. If this protocol is effective it will be an answer of translational empirical research into effective actions (Gruber et al., 2016), presenting one way for optimizing sleep hygiene practices with children by using a serious game, preventing a series of impacts of inappropriate sleep on health, cognition and the risk at psychiatric conditions.

Poor and insufficient sleep is still an “invisible risk” and many parents and professionals underestimate children’s sleep problems. Conversely, recommendations of a sleep routine and a consistent sleep schedule are currently associated with improved sleep onset-latency and sleep consolidation, while delayed bedtime reduces the number of hours available to sleep, since children are not allowed to sleep in the morning due to school or the parent’s schedules. This recurring delay and consequent sleep loss impacts on mood, cognition, appetite and in prolonged daytime naps (Komada et al., 2011; Kitamura et al., 2015).

With the improvements in technology there is also an increase in the use of media for entertainment and work. This context is no different for children and some specialized organizations are beginning to create recommendations on limits to the use of technologies by age, however, there are still no recommendations that also consider the content and purpose of the activity developed in electronics devices (Baranowski et al., 2016; Van Rooij et al., 2017).

Presence of electronic media in the bedroom (e.g., television, video game, and computer) and the use of them next to bedtime are related to late bedtime and decreased sleep duration. In addition, children that sleep next small screens (e.g., smartphones) relate impacts in perception of insufficient rest, probably caused by waking up due to notifications on the small screen (Cain and Gradisar, 2010; Falbe et al., 2015). The opposite is also true, and children that do not have electronics in the bedroom report more hours of sleep. Other factors related to the bedroom are also relevant, such as providing a comfortable bedroom and conditions to sleep, but this is challenging for families of low SES (Chung et al., 2014; Bagley et al., 2015).

Our serious game includes recommendations in sleep environment, but just the game is not enough to help children in families with low-incomes. Provide conditions, such as quiet and dark bedroom and comfortable bed, depends on the neighborhood that the families live and their incomes. Thus, subsequent interventions should include actions to help these families provide a comfortable bedroom, such as Time’s Beds for Kids in EUA (Mindell et al., 2016).

It stands-out that in Brazil did not exist, in our knowledge, any questionnaire adapted and validated to evaluate the chronotype of children with ages between 7 and 8 years, as different types have influences on the presence of sleep disorders and accentuate inadequate sleep habits (Jafar et al., 2017), so chronotype influence was not assessed in this protocol.

Other pitfall that could be noted, is the consideration that parent’s reports may be influenced by social desirability, indicating good sleep hygiene practices instead of the real situation. Another important challenge is to promote parental involvement, since previous researches indicated a decrease in participation over the evaluation periods (Jones et al., 2013; Wilson et al., 2014; Mindell et al., 2016). In this way, we proposed follow-up meetings with parents to minimize this decrease on participation.

Although in this protocol efficacy measures in evaluation will be limited to sleep habits and patterns, the promotion of optimal sleep has consequences in multiples domains (e.g., cognitive, health, temperament) that are sometimes completely forgotten in sleep programs interventions (Blunden, 2017). Thus, future studies should include other outcomes to evaluate efficacy, such as cognitive and academic performance.

Therefore, the confluence of these factors, problems related to sleep and insufficient sleep, and the absence of adequate intervention or treatment are associated in the short and long term with negative impacts on overall health and performance. In this way, sleep hygiene practices may promote healthy sleep in children, which could influence in health outcomes and decrease the social burden of treatments. Our serious game could be applied in clinical, educational settings to promote good sleep hygiene in children and prevent these negative consequences. In addition, “Perfect Bedroom” could be applied at home with supervision of parents becoming a approach less costly compared to others cognitive and behavioral interventions used to improve sleep in children, also, reducing burdens to treatments of negative consequences of inappropriate sleep on children’s normal developmental.

## Author Contributions

ML and KA contributed to the study on the original idea, design, and proposed analysis. ML wrote the first draft of the manuscript and all authors approved the final manuscript.

## Conflict of Interest Statement

The authors declare that the research was conducted in the absence of any commercial or financial relationships that could be construed as a potential conflict of interest.

## Footnotes

1 http://www.graphpad.com/quickcalcs/index.cfm
